# A Rapid Segmentation-Insensitive “Digital Biopsy” Method for Radiomic Feature Extraction: Method and Pilot Study Using CT Images of Non–Small Cell Lung Cancer

**DOI:** 10.18383/j.tom.2016.00163

**Published:** 2016-12

**Authors:** Sebastian Echegaray, Viswam Nair, Michael Kadoch, Ann Leung, Daniel Rubin, Olivier Gevaert, Sandy Napel

**Affiliations:** 1 Department of Electrical Engineering, Stanford University, Stanford, California;; 2 Department of Radiology, Stanford University School of Medicine, Stanford, California;; 3 Department of Medicine, Stanford University School of Medicine, Stanford, California; and; 4 Canary Center for Cancer Early Detection, Stanford University, Stanford, California

**Keywords:** radiomics, segmentation, CT, image processing, medical imaging, quantitative imaging

## Abstract

Quantitative imaging approaches compute features within images' regions of interest. Segmentation is rarely completely automatic, requiring time-consuming editing by experts. We propose a new paradigm, called “digital biopsy,” that allows for the collection of intensity- and texture-based features from these regions at least 1 order of magnitude faster than the current manual or semiautomated methods. A radiologist reviewed automated segmentations of lung nodules from 100 preoperative volume computed tomography scans of patients with non–small cell lung cancer, and manually adjusted the nodule boundaries in each section, to be used as a reference standard, requiring up to 45 minutes per nodule. We also asked a different expert to generate a digital biopsy for each patient using a paintbrush tool to paint a contiguous region of each tumor over multiple cross-sections, a procedure that required an average of <3 minutes per nodule. We simulated additional digital biopsies using morphological procedures. Finally, we compared the features extracted from these digital biopsies with our reference standard using intraclass correlation coefficient (ICC) to characterize robustness. Comparing the reference standard segmentations to our digital biopsies, we found that 84/94 features had an ICC >0.7; comparing erosions and dilations, using a sphere of 1.5-mm radius, of our digital biopsies to the reference standard segmentations resulted in 41/94 and 53/94 features, respectively, with ICCs >0.7. We conclude that many intensity- and texture-based features remain consistent between the reference standard and our method while substantially reducing the amount of operator time required.

## Introduction

Quantitative features computed from medical images have been investigated for use in computer-aided diagnosis (1, 2), computer-aided detection (3, 4), and radiomics (5–7). Although these image features have the potential to provide consistent descriptors of the object being analyzed, segmentation of the volume of interest (VOI) is the necessary first step for obtaining these values. Manual segmentation of tumors in 2-dimensional (2D) computed tomography (CT)-images is labor-intensive and time-consuming (8, 9), and even more onerous when segmenting 3-dimensional (3D) volumes. The literature contains several automatic and semiautomatic image segmentation algorithms (10–18). Although these algorithms reduce the time taken to segment a tumor (19, 20), they do not always achieve accurate or consistent results (21); therefore, all segmentations must be reviewed and possibly edited, which, in turn, requires additional time and introduces variability.

The typical approach to using the radiomics features to build predictive models involves computing a large number of image features from within a VOI. Because many of these computed features are correlated, one of the several machine learning methods (22, 23) can be used to select a subset of nonredundant informative features that can be combined in the model (24–26). We hypothesized that a set of features required for a robust predictive model could be extracted without requiring accurate or precise segmentation of the tumor boundary. The first step in testing this hypothesis involves determining which features extracted from such a segmentation are consistent with a segmentation that attempts to capture the tumor margin. Therefore, we developed a new method, which we call “digital biopsy,” in which a human annotator is asked to capture the heterogeneity of the tumor without carefully segmenting the tumor boundary, by “painting” the inside of the tumor using a suitable tool. Then, we analyzed the stability of the intensity and texture features of segmentations of the entire tumor computed from these digital biopsies compared with those computed semiautomatically by calculating their intraclass correlation coefficient (ICC) (27, 28). Because we did not expect the tumor shape or margin to be captured by these digital biopsies, we did not compute ICCs for such features.

In doing so, we introduced a new segmentation paradigm, in which the expert focuses on capturing the heterogeneity of the lesion instead of the tumor boundary. In a preliminary study of 100 patients with preoperative CT scans of proven non–small cell lung cancer, we show that multiple image features that characterize the intensity and texture of lung nodules are consistent with the same features that could be obtained using detailed segmentations, while obtaining an order of magnitude reduction in human operator time.

## Materials and Methods

### Data

Following institutional review board approval by Stanford University's Research Compliance Office, that waived the requirement for written informed consent, we selected the first 100 subjects (male, 75; mean age, 70 years) who were part of a radiogenomics cohort obtained from a larger database of patients referred to Stanford University Medical Center with diagnosed non–small cell lung cancer and had CT scans before surgical resection. Scanners (GE Medical Systems, Waukesha, WI; Siemens, Erlangen, Germany; Toshiba, Otawara, Japan; and Phillips, Andover, MA, with 120 kVp), with current and section thickness ranging from 25 to 697 mA and 0.625 to 3 mm, respectively, were used to acquire scans. The tumors ranged in size from 0.37 to 306 cm^3^ and in attenuation from ground glass to solid. All scans were performed between April 2008 and October 2014.

### Semiautomated—Manual Segmentations

For this study, we used an in-house segmentation algorithm (21) under supervision of a fellowship-trained thoracic radiologist with 23 years of experience. For each subject, the radiologist first identified the tumor in the CT scan volume and chose a small “seed circle” to initialize the segmentation algorithm, which then derived a 3D VOI containing the voxels within the tumor, which was then superimposed on the CT slices using ePAD, an open-source annotation tool (29, 30). Finally, the radiologist spent between 5 and 45 minutes manually editing each VOI using ePAD's paintbrush tool on 2D cross-sections to correct what he perceived as local over- and/or under-segmentation.

### Digital Biopsies

**Figure 1.**
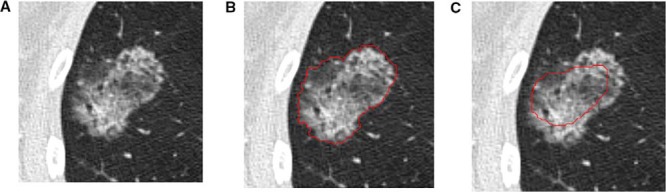
Cross-section through part-solid nodule in the right upper lobe (A), and its intersection with the reference standard 3-dimensional (3D) segmentation (B), and the 3D digital biopsy (C).

A nonradiologist human reader with 30 years of experience with medical image processing and analysis (10 years specifically with CT scans of patients with lung cancer) independently viewed each series and created a digital biopsy using the same paintbrush tool used by the radiologist on 2D cross-sections of the tumor. Using the reference standard for each of the 100 subjects as a guide, this reader marked a contiguous section of the tumor volume for inclusion as he scrolled through the 2D cross-sections in the image series. The reader was instructed to capture the gray-scale heterogeneity of the tumor and to not be concerned with capturing the detail of the boundary. We gave no additional instructions about how to segment the sections or which section to select. We tracked the time required to create each digital biopsy and stored each of them to compare with the radiologist's reference standard. Figure 1 shows a central section through one of the nodules and the reference standard 3D segmentations and 3D digital biopsy.

### Simulating Multiple Readers

**Figure 2.**
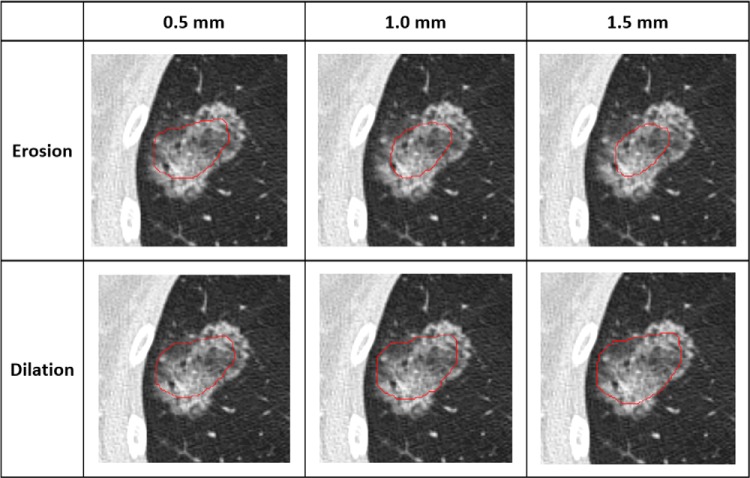
Cross-sections of digital biopsies obtained by applying morphological operations to the manual digital biopsy shown in Figure 1. The first and second rows show erosions and dilations, respectively.

As the creation of digital biopsies is subjective and therefore prone to intra- and inter-reader variation, we simulated additional digital biopsies using the morphological procedures of erosion and dilation (31). These were achieved using multiple spherical structuring elements with radii ranging from 0.5 to 1.5 mm inclusive, with an interval of 0.5 mm between the radii, for a total of 3 erosions and 3 dilations. The erosions simulated more conservative additional readers, while the dilations simulated more aggressive additional readers. Dilations were not constrained to stay within any region and therefore may have led to the digital biopsy going beyond the tumor borders. To avoid splitting the tumor into multiple portions during erosion, we followed each with a morphological closing procedure. Figure 2 shows an example of the erosions and dilations from the same nodule and digital biopsy shown in Figure 1.

### 3D Image Features

There are many algorithms in the literature that extract quantitative features from VOIs (32–34) within a CT scan. These algorithms can be categorized as measuring intensity, shape, and margin or textures' characteristics. Intensity features express statistics of the pixel values within a VOI. Shape features describe the boundary of the VOI. Margin features characterize the transition between the intensity values inside the VOI and the values surrounding it. Finally, texture features measure the spatial distribution of pixel intensities inside the VOI. For this study, we only computed intensity and texture features (a total of 94 features) because our digital biopsies, by definition, do not attempt to capture the shape or margins of the tumors. Appendix 1 provides a list of the specific intensity and texture features that we computed, with references to the literature as needed.

### Metrics

#### Overlap.

To analyze the agreement between *i* VOIs in a given series, k, we define overlap as the ratio between their intersections and their unions as follows:

$$O_{k}=\frac{\cap_{i}VOI_{i}}{\cup_{i}VOI_{i}}$$

### Feature Consistency

We used the ICC to measure the consistency of the features extracted for each segmentation looking across patients, readers, cores, and sections. The ICC describes how members from the same group resemble each other and has often been used to quantify the consistency of measurements made by different experts (35, 36). A high ICC shows that a feature is consistent across multiple measurements. There are multiple algorithms in the literature to calculate ICC (37); for this study, we used the A-1 method, also known as criterion-referenced reliability, which is expressed as follows:

$$ICC=\frac{MS_{R}-MS_{E}}{MS_{R}+\left(k-1\right)MS_{E}+\frac{k}{n}\left(MS_{C}-MS_{E}\right)}$$

where MS_R_ is the mean square for rows (observations), MS_E_ is the mean square error, and MS_C_ is the mean square for columns (segmentations). n and k represent the total number of rows and columns, respectively. In our study, rows represent each study where features were extracted. Columns represent the different segmentations, which can be provided by the reference standard segmentations, digital biopsies, or the morphological operations. We used this method, as it measures the degree of absolute agreement taking into consideration the systematic variations between methods.

## Results

### Digital Biopsies: Time to Obtain and Volume Overlap with Reference Standard

**Figure 3.**
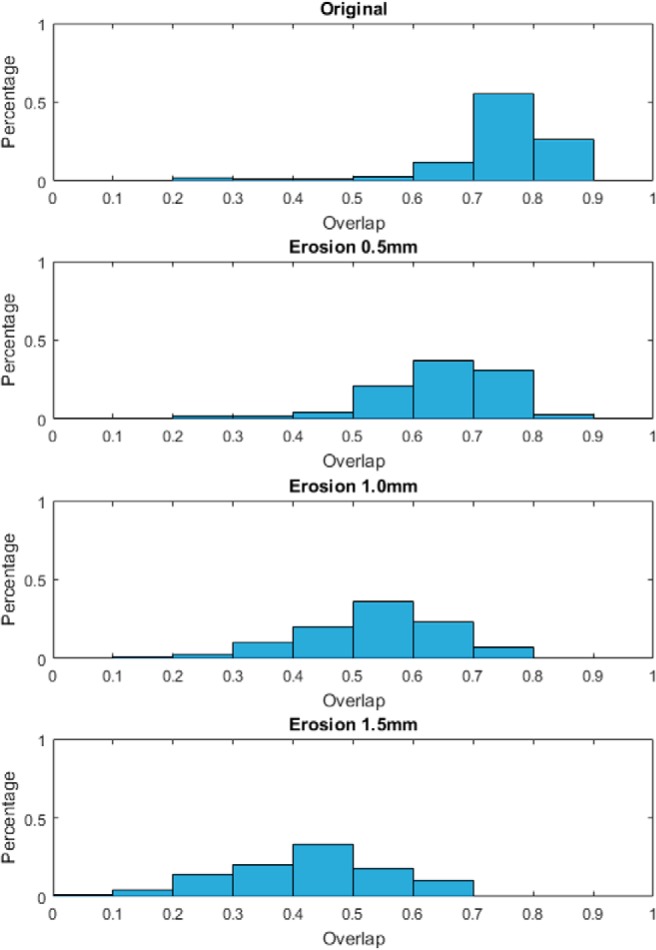
Distribution of the overlap of the reference standard segmentation and in order from top to bottom: the original biopsy, 0.5-mm erosion, 1.0-mm erosion, and 1.5-mm erosion. Statistics regarding the distributions are shown in Table 1.

**Figure 4.**
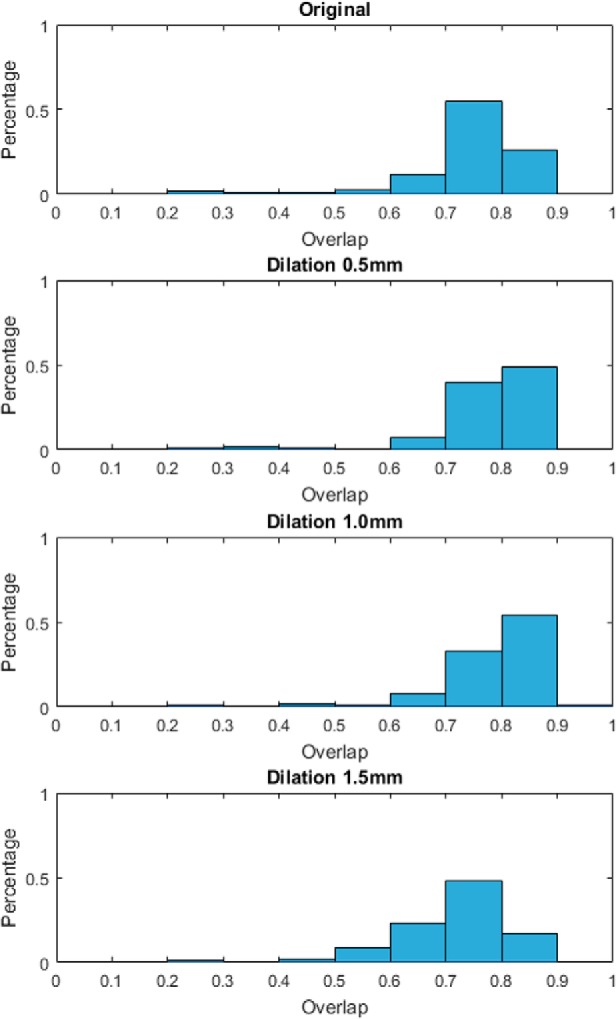
Distribution of the overlap of the reference standard segmentation and in order from top to bottom: the original biopsy, 0.5-mm dilation, 1.0-mm dilation, and 1.5-mm dilation. Statistics regarding the distributions are shown in Table 1.

Using ePAD's paintbrush tool, the reader averaged 171 seconds, with a median of 132 seconds, a standard deviation of 152 seconds, and a range 25 to 900 seconds, to create a digital biopsy. Table 1 shows the mean and standard deviation, median, minimum, and maximum of the volume overlap of the reference standard with the digital biopsies with the 3 erosions and the 3 dilations. One can see that the volume overlap decreases with erosion of the digital biopsies, as expected, and that it can also decrease with the dilation of the digital biopsies as the dilated volumes grow bigger than the digital biopsies. Figure 3 and Figure 4 show the distribution of these overlaps as a function of erosion and dilation, respectively, of the digital biopsies. Erosions caused the distribution to shift to the left (lower overlap), while dilations caused an initial shift to the right (higher overlap) at 0.5 mm of dilation, with a shift to the left as larger dilations expanded the volumes to exceed the nodule borders in many cases.

**Table 1.** Overlap Between Digital Biopsies and the Reference Standard Segmentation

Method	Size	Mean (%)	SD (%)	Median (%)	Minimum (%)	Maximum (%)
Original digital biopsies	None	74.04	10.77	76.65	25.26	85.23
Erosions	0.5 mm	64.46	11.27	67.11	20.94	82.81
	1.0 mm	53.04	12.03	54.87	13.63	75.52
	1.5 mm	42.33	13.11	42.61	8.47	69.61
Dilations	0.5 mm	77.73	10.38	79.49	27.11	88.58
	1.0 mm	78.21	9.66	80.36	28.22	90.94
	1.5 mm	71.43	10.22	73.34	28.55	89.17

Abbreviation: SD, Standard deviation.

### Digital Biopsies: Agreement of Features With Those Obtained Using the Reference Standard Segmentations

**Figure 5.**
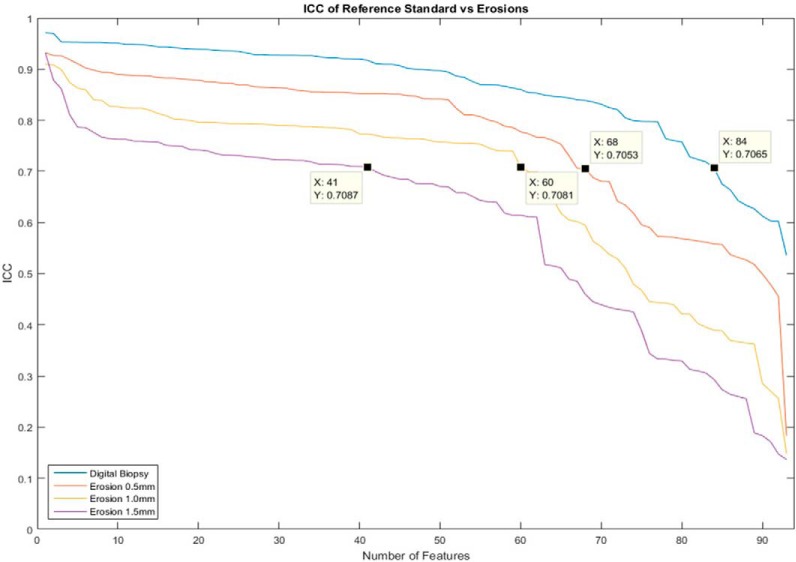
The intraclass correlation coefficient (ICC) curves for the features extracted from the digital biopsies and each of the morphological erosions compared with their reference standard segmentation. The features are organized in the descending order by their ICC value. Each line has been marked to indicate the number of features, with ICC > 0.7.

**Figure 6.**
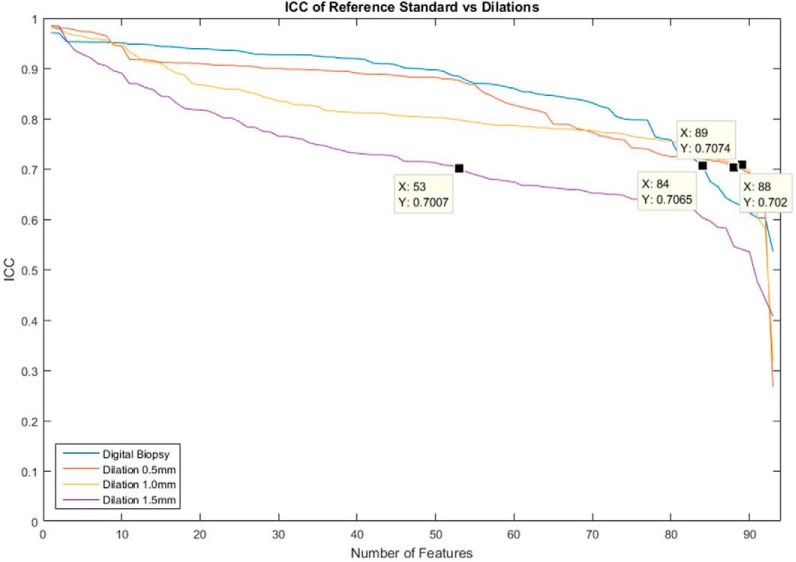
The ICC curves for the features extracted from the digital biopsies and each of the morphological dilations compared with their reference standard segmentation. The features are organized in the descending order by their ICC. The features are organized in the descending order by their ICC. Each line has been marked to indicate the number of features, with ICC > 0.7.

We obtained the ICC score for each of the features extracted from the digital biopsies and their erosions and dilations compared with the features extracted from the reference standard. Figure 5 and Figure 6 plot the ICC scores, with features ordered from the highest ICC on the left to the lowest ICC on the right for each curve separately, for the erosions and dilations of the digital biopsies, respectively. These figures show that 84/94 features computed using the original digital biopsies have excellent agreement (ICC > 0.7) (37–39), with the same features computed using the reference standard segmentations. Moreover, eroding the digital biopsies continued to produce many features showing excellent agreement with those computed using the reference standard segmentations (68/94, 60/94, and 41/94 for 0.5 mm, 1.0 mm, and 1.5 mm erosions, respectively). Similarly, dilation resulted in many features showing excellent agreement with those computed using the reference standard segmentations (88/94, 89/94, and 53/94 for 0.5 mm, 1.0 mm, and 1.5 mm dilations, respectively). Table 2 shows the number of features above several other thresholds of agreement (ICCs of 0.6 through 0.9), revealing that many features are insensitive to the exact borders of the segmentation. Appendix 2 contains a ranked list showing the most robust features across all 7 experiments.

**Table 2.** Features Above Thresholds of Agreement

ICC	Original	Erosion			Dilation		
		0.5 mm	1.0 mm	1.5 mm	0.5 mm	1.0 mm	1.5 mm
>0.9	47	6	2	1	27	15	8
>0.8	74	56	18	4	64	51	24
>0.7	84	68	60	41	88	89	53
>0.6	93	74	67	62	92	91	84

The number of features presented in the table are out of 94 that presented ICC > 0.9, > 0.8, > 0.7, and > 0.6 in each of the digital biopsies and its morphological modifications when compared with the features extracted from the reference standard segmentation. Appendix 2 names and ranks the individual features with the highest ICCs across all 7 digital biopsy variations.

**Figure 7.**
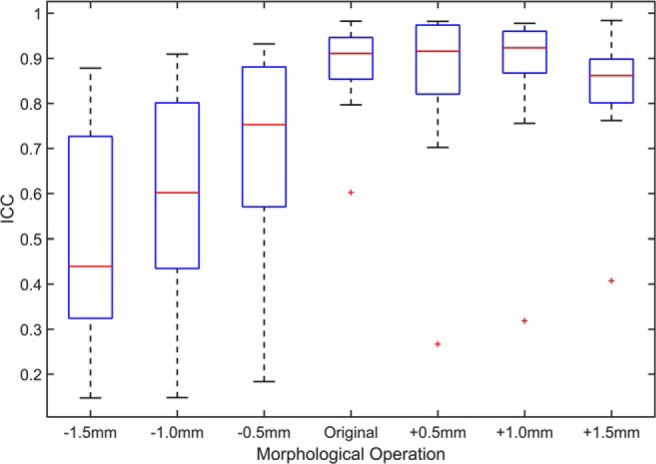
Boxplot of ICC of intensity features for original digital biopsies and their erosions/dilations compared with the reference standard segmentations. The Y-axis shows the ICC score and the X-axis is the morphological operation, with “−” and “+” representing that the segmentation underwent erosion and dilation, respectively.

**Figure 8.**
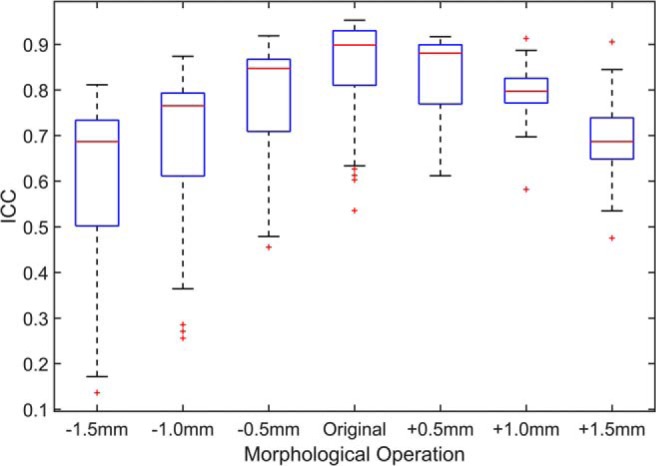
Boxplot of ICC of texture features for original digital biopsies and their erosions/dilations compared with the reference standard segmentations. The Y-axis shows the ICC score and the X-axis is the morphological operation, with “−” and “+” representing that the segmentation underwent erosion and dilation, respectively.

Figure 7 and Figure 8 show the distribution of ICC scores for intensity and texture features, respectively, and how these distributions change as a function of erosion and dilation. Figure 7 shows that intensity features maintain high ICCs under dilation, but fall off under erosion. Figure 8 shows that the ICCs of texture features decrease under both procedures, although, as was the case for intensity features, more strongly under erosion.

## Discussion

The radiomics methods, in widespread development, compute many (sometimes hundreds of) image features from VOIs or regions of interest (ROIs) in radiological images and link these features to clinical data, such as response to treatment, survival, and gene expression (5, 15, 25, 26, 40, 41). Once these features are computed, they can be used to build predictive models. Several studies have shown that the collection of images' features computed are highly correlated (25, 41, 42), and the resulting models usually are based on a small subset of the robust and independent computed features. Most often, ROIs are selected using segmentation algorithms and/or manual delineation. Although several software packages have been developed by academic institutions (10–15, 21, 43–48) and commercial vendors that offer automated segmentation of lung nodules in chest CT scans, in our experience, none is foolproof; on the contrary, each segmentation must be reviewed for quality control and perhaps edited. Thus, we wondered if a set of features could be computed from an easier-to-obtain ROI that shows consistency with features from full quality-controlled segmentation. The median time and maximum time required to obtain a digital biopsy were 132 and 900 seconds, respectively. This is an order of magnitude that is faster than our reference standard procedure, wherein a trained radiologist had to inspect and modify the semiautomated segmentation to trace the tumor boundary, which required anywhere from 600 to 2700 seconds (5–45 minutes) per case. Further, despite the lack of precise overlap of our digital biopsies to the reference standard, we have shown many features that remain highly consistent. Table A2 (Appendix 2) lists the many robust features we have found that are important descriptors of tumoral heterogeneity, and that have been used in many radiomics studies in several cancer imaging scenarios (5–7, 15, 22, 41, 49).

Although our digital biopsies were much faster to obtain, we acknowledge that for this technique to be used in practice, it would have to be even faster. We have identified multiple addressable factors that limited our speed in obtaining them. First, this was the first time this expert used this tool for this purpose. It is likely that familiarity would result in an increase in speed. Second, although ePAD is a powerful Digital Imaging and Communications in Medicine (DICOM) viewer and annotation tool, it was not created with this use in mind; painting was accomplished 1 transaxial section at a time, and the reader had to navigate through multiple menus to change the ePAD's brush size to gain efficiency as the cross-sectional area on each section changed. We are confident that a tool that works on multiple planes and has streamlined operations could reduce the required time by another order of magnitude, thus reducing the median time to under 1 minute per tumor.

Obtaining fast digital biopsies is only useful if the features that are being extracted are consistent among operators and methods. As shown in Table 2, 89% (84/94) of the features remain highly consistent (ICC > 0.7) between the reference standard segmentation and the original digital biopsies, even while the digital biopsy only segments 74% of the original tumor (Table 1). This is promising, in that it shows that we are able to capture the same information using a less precise but faster segmentation. This stability remains mainly present as we dilate and erode our digital biopsies with a spherical structuring element to up to 1.5 mm in radius, representing readers that are more or less conservative with their segmentations, as shown in Figures 5 and 6. These erosions and dilations, simulating additional readers, also produced a large number of features with ICC > 0.7. The discrepancies between erosions and the original digital biopsies are mainly caused by the change of point statistics (eg, extrema), and the loss of higher wavelength textures. In contrast, discrepancies between dilations and the original biopsies can be additionally caused by inclusion of voxels beyond the periphery of the tumor, which can result in some strong texture responses (eg, at the boundary between the tumor and the air in the lung or an adjacent chest wall). We can observe these discrepancies in both Figures 7 and 8, where we see that the intensity features remain mostly stable under dilation, while stability is reduced by erosion, and that texture features' consistency is reduced under both procedures. However, in all circumstances, the majority of features remain consistent with those obtained by our reference standard segmentation.

### Limitations

One limitation is that we only used one manual digital biopsy per subject; different readers would not necessarily result in the same VOI. We attempted to overcome this limitation by generating multiple simulated biopsies by morphologically altering our reader's segmentations. As we have shown, multiple texture and intensity features remain highly stable across the simulated segmentations; therefore, we expect similar results if we were to include multiple readers, as long as their mean volume overlap with the tumor itself is at least 75%. However, future studies with multiple users are required to validate that intra- and inter-reader variation of ICCs of features derived from these digital biopsies is acceptable. Other painting strategies may also be useful, such as creating multiple small digital biopsies per tumor, and remain to be investigated.

Second, to obtain a preliminary comparison of the segmentation time improvement, we compared the time required by a radiologist to carefully adjust segmentation boundaries to that required by a nonradiologist to acquire the digital biopsies, resulting in an order of magnitude speed advantage for the digital biopsies. Although comparing a radiologist with a nonradiologist could confound the comparison, it is not clear that either participant would have a speed advantage over the other given the different tasks. Future studies should compare multiple radiologists and trained nonradiologists (who ideally would be preferred for this task on the basis of cost).

Third, as part of our experimental design, we have eliminated all boundary and shape features from our study. The literature has reports that boundary and shape are important markers in characterization of certain cancers (50–52). Our proposed method for segmenting does not capture these features, and it remains to be shown that the intensity and texture features that we do capture are sufficient to build strong predictive models. It is also possible that complementing the features obtained via digital biopsy with a small set of easy-to-obtain semantic features (eg, “spiculated,” “lobulated,” “pleural attachment,” and “poorly defined margins”) could strengthen the model at little cost; this too, remains, for future evaluation.

Fourth, we acknowledge that the reader who provided the original digital biopsies used the reference standard as a guide to locate each tumor, information that would not be available in general. It is therefore possible that performance with unguided digital biopsies would be different than that reported here. Although our experiments simulating additional readers with morphological operations provide some reassurance, performance with unguided digital biopsies by trained readers should be evaluated in the future.

Fifth, in this study, we have only focused on the stability of the features and have not proven, nor intended to prove, that these features correlate with any clinical or outcomes data. As Aerts (24) and others have shown, a first step in building these models is to find features that are robust, that is, insensitive to segmentation. We have shown that our digital biopsy technique is robust in the context of CT for lung cancer, and it remains to show this in other cancer types and imaging scenarios. Discovering relationships and building models linking these features to disease is the next step. Rapidly obtained digital biopsies may prove quite useful in this endeavor.

Finally, our study only addresses the stability of the features to changes in tumor segmentation. However, it is well known that CT acquisition and reconstruction parameters and conditions can also affect quantitative feature values (53–57). Future studies that compare quantitative image features acquired at different points in time must be aware of this possibility and control for this effect.

In conclusion, we have proposed a new paradigm for selecting a VOI for the radiomics analysis that captures the heterogeneity of a given lesion in 3D. This method is 1 or 2 orders of magnitude faster than semiautomated segmentations, which has remained the dominant strategy because completely automatic segmentation has not been shown. We have shown that the texture- and intensity-based features extracted in this way are robust to morphological transformations and remain highly correlated with those from curated segmentations and, therefore, we think that the use of digital biopsies will accelerate the potential of researchers to develop, and for clinicians to use, quantitative imaging methods to characterize cancer in medical images.

## Appendix 1

### Intensity Features

To quantify the intensity characteristics of the volume, we extracted all the classical statistical descriptors such as mean, variance, kurtosis, skewness, entropy, maximum, and minimum from inside the VOI. These features summarize the global information of the intensity distribution inside the VOI without considering local differences.

### Texture Features

To characterize local changes in intensity, we computed the Haralick features. The Haralick features are measurements taken from a gray-level co-occurrence matrix (GLCM) (58–60). A GLCM is defined as the distribution of co-occurring values in an image at a fixed offset. This matrix is defined by the following equation:

$$C\left(i,j,\Delta x,\Delta y,\Delta z\right)=\overset{N}{\underset{p=1}{\sum }}\overset{M}{\underset{q=1}{\sum }}\overset{O}{\underset{r=1}{\sum }}\{\frac{1,}{0,}\frac{\begin{array}{c} if\;I\left(p,q,r\right)=i\;and\\ I(p+\Delta x,q++\Delta y,r++\Delta x)=j \end{array}}{\;otherwise}$$

where i and j are the row and columns indices of the GLCM, respectively. Δx, Δy, and Δz are the fixed offsets in the three axes of the volume. I(p,q,r) is the gray level at point (p,q,r) and N,M,O, are the sizes of the volume in each dimension. A set of statistics, shown with their references in Table A1, is then computed from the GLCM. To obtain rotation-invariant features, we calculate the Haralick features in 13 directions, aggregate them, and report their mean and standard deviation.

**Table A1.** List of Haralick Features Extracted from the GLCM

Features	References
Energy	(59, 61)
Contrast	(59, 61)
Sum of Means	(61)
Cluster Tendency	(59)
Entropy	(59)
Homogeneity	(59)
Inertia	(58, 59)
Max Probability	(59)
Correlation	(59, 61)
Variance	(61)
Cluster Shade	(59)
Inverse Variance	(58, 59)

Abbreviation: GLCM, gray-level co-occurrence matrix.

Details of the implementation of each of these features can be found in the references mentioned alongside the features.

## Appendix 2

In Table A2, we count how many times each feature was over a certain ICC value over each of the 7 experiments. (Reference Standard vs Digital Biopsy and 0.5 mm, 1.0 mm, 1.5 mm erosions and dilations). Features that did not have an ICC > 0.7 in any of the experiments are obviated. Features that consistently have high ICC across all experiments indicate to be more robust to changes in their contour than features with rapidly changing ICC.

**Table A2.** Features of Important Descriptors of Tumoral Heterogeneity

Feature Name	ICC > 0.7	ICC > 0.8	ICC > 0.9
“Haralick D=3mm std sum of means”	7	7	3
“Intensity Entropy”	7	6	5
“Intensity Mean”	7	6	3
“Intensity Median”	7	6	3
“Intensity Trimmed Mean (25%)”	7	6	3
“Haralick D=1mm std energy”	7	6	1
“Haralick D=2mm std entropy”	7	6	1
“Haralick D=1mm std max probability”	7	5	4
“Haralick D=1mm std entropy”	7	5	2
“Haralick D=1mm mean cluster tendency”	7	5	1
“Haralick D=1mm std sum of means”	7	5	1
“Haralick D=2mm std sum of means”	7	5	1
“Haralick D=3mm std cluster shade”	7	5	1
“Haralick D=2mm std max probability”	7	5	0
“Haralick D=2mm mean cluster tendency”	7	4	1
“Haralick D=3mm mean cluster tendency”	7	4	1
“Haralick D=2mm std cluster shade”	7	4	1
“Haralick D=3mm std contrast”	7	4	1
“Haralick D=3mm std inertia”	7	4	1
“Haralick D=2mm std energy”	7	4	0
“Haralick D=1mm std variance”	7	3	0
“Haralick D=2mm std variance”	7	1	0
“Intensity Under −291 HU Percentage”	6	5	3
“Intensity Over −291 HU Percentage”	6	5	3
“Haralick D=1mm mean variance”	6	5	2
“Haralick D=2mm mean variance”	6	5	2
“Haralick D=3mm mean variance”	6	5	2
“Haralick D=3mm std max probability”	6	5	0
“Haralick D=2mm std contrast”	6	4	2
“Haralick D=2mm std inertia”	6	4	2
“Haralick D=1mm mean cluster shade”	6	4	1
“Haralick D=2mm mean cluster shade”	6	4	1
“Haralick D=1mm std cluster shade”	6	4	1
“Haralick D=2mm std cluster tendency”	6	4	1
“Haralick D=3mm std cluster tendency”	6	4	1
“Haralick D=3mm mean cluster shade”	6	4	0
“Haralick D=1mm std contrast”	6	3	2
“Haralick D=1mm std inertia”	6	3	2
“Haralick D=1mm mean entropy”	6	3	0
“Haralick D=2mm mean entropy”	6	3	0
“Haralick D=3mm std variance”	6	3	0
“Haralick D=3mm mean energy”	6	2	0
“Haralick D=3mm mean max probability”	6	2	0
“Haralick D=1mm mean energy”	6	1	0
“Haralick D=2mm mean energy”	6	1	0
“Haralick D=2mm mean max probability”	6	1	0
“Haralick D=3mm std entropy”	5	5	1
“Intensity Skewness”	5	4	2
“Intensity Min”	5	4	2
“Haralick D=1mm mean contrast”	5	4	2
“Haralick D=1mm mean inertia”	5	4	2
“Haralick D=1mm std cluster tendency”	5	4	1
“Haralick D=2mm mean contrast”	5	3	2
“Haralick D=2mm mean inertia”	5	3	2
“Haralick D=3mm mean contrast”	5	3	1
“Haralick D=3mm mean inertia”	5	3	1
“Haralick D=1mm std homogeneity”	5	3	1
“Haralick D=2mm std homogeneity”	5	3	1
“Haralick D=3mm mean entropy”	5	3	0
“Haralick D=3mm std homogeneity”	5	3	0
“Haralick D=1mm mean max probability”	5	1	0
“Intensity Mean Absolute Difference”	4	4	1
“Intensity Standard Deviation”	4	4	0
“Intensity Interquartile Difference “	4	2	1
“Intensity Range”	4	2	0
“Intensity Max”	4	2	0
“Haralick D=3mm std energy”	4	1	0
“Haralick D=1mm mean sum of means”	4	0	0
“Haralick D=2mm mean sum of means”	4	0	0
“Haralick D=3mm mean sum of means”	4	0	0
“Haralick D=1mm mean correlation”	3	1	0
“Intensity Kurtosis”	3	0	0
“Haralick D=1mm mean homogeneity”	3	0	0
“Haralick D=1mm mean inverse variance”	3	0	0
“Haralick D=2mm mean homogeneity”	3	0	0
“Haralick D=2mm mean inverse variance”	3	0	0
“Haralick D=3mm mean homogeneity”	3	0	0
“Haralick D=3mm mean inverse variance”	3	0	0
“Haralick D=2mm std correlation”	3	0	0
“Haralick D=1mm std correlation”	2	1	0
“Haralick D=3mm std inverse variance”	2	1	0
“Haralick D=2mm mean correlation”	2	0	0
“Haralick D=3mm mean correlation”	2	0	0
“Haralick D=2mm std inverse variance”	2	0	0
“Intensity Harmonic Mean”	1	1	1
“Intensity Mode”	1	1	0
“Haralick D=3mm std correlation”	1	0	0

Abbreviation: ICC, Intra-class correlation.

The features mentioned were computed for each of the 7 digital biopsies (original, 3 erosions, and 3 dilations) and the number of times their ICC compared with the reference standard was higher than 0.9, 0.8, and 0.7, ranked by ICC. Features that never scored >0.7 are not shown.
